# Multi-walled carbon nanotubes-induced alterations in microRNA *let-7* and its targets activate a protection mechanism by conferring a developmental timing control

**DOI:** 10.1186/s12989-017-0208-2

**Published:** 2017-07-20

**Authors:** Li Zhao, Hanxiao Wan, Qizhan Liu, Dayong Wang

**Affiliations:** 1 Key Laboratory of Environmental Medicine Engineering in Ministry of Education, Medical School, Southeast University, Nanjing, 210009 China; 2 School of Public Health, Nanjing Medical University, Nanjing, 211166 China

**Keywords:** *let-7*, Multi-walled carbon nanotubes, Developmental timing, Nanotoxicity, *Caenorhabditis elegans*

## Abstract

**Background:**

Multi-walled carbon nanotubes (MWCNTs) have been produced and applied for diverse purposes. MWCNTs can potentially cause the adverse effects on organisms. MicroRNA *let-7* and its targets of HBL-1 and LIN-41 play a central role in regulating developmental timing by acting as a developmental switch. The sequence of *let-7* and the underlying mechanisms for *let-7* in the control of developmental timing are conserved among different species. In this study, we investigated the potential effect of MWCNTs exposure on the molecular basis for developmental timing mediated by *let-7* and its targets of HBL-1 and LIN-41.

**Results:**

MWCNTs exposure decreased *let-7* expression, and increased expressions of *hbl-1* and *lin-41*. *let-7* mutant was resistant to MWCNTs toxicity, whereas *hbl-1* or *lin-41* mutant was susceptible to MWCNTs toxicity. *hbl-1* or *lin-41* mutant suppressed the resistance of *let-7* mutant to MWCNTs toxicity. HBL-1 acted upstream of TIR-1, an adaptor protein, and SYM-1, a protein containing leucine-rich repeats, and ALG-1 and ALG-2, two RDE-1 proteins related to RNA interference (RNAi) pathway, acted upstream of LIN-41 to regulate MWCNTs toxicity. Moreover, we identified a feedback loop between *let-7* and its targets of HBL-1 and LIN-41 in the regulation of MWCNTs toxicity. The observed increase in *let-7::GFP* expression in MWCNTs exposed nematodes with mutation of *hbl-1* or *lin-41* implied that a feedback mechanism may exist to strengthen the function of *let-7* suppression in protecting the animals from MWCNTs toxicity.

**Conclusions:**

Our results demonstrate the protection function of *let-7* suppression for animals from MWCNTs toxicity by conferring a robust developmental timing control. Our results highlight the importance of molecular basis for developmental timing in influencing MWCNTs toxicity.

**Electronic supplementary material:**

The online version of this article (doi:10.1186/s12989-017-0208-2) contains supplementary material, which is available to authorized users.

## Background

Multi-walled carbon nanotubes (MWCNTs), one member of the carbon-based engineered nanomaterials (ENMs), have numerous unique physicochemical properties, such as high electrical conductivity and thermal conductivity [1]. MWCNTs have been produced in bulk for diverse purposes, and can be potentially used in several fields, including drug delivery, heterogeneous catalysis to optoelectronic device development, and environmental remediation [2–4]. With the increase in MWCNTs manufacture, the exposure possibility of human and environmental organisms to MWCNTs is also increased [5, 6]. A series of in vitro and in vivo studies have demonstrated several aspects of MWCNTs toxicity on organisms, such as the pulmonary toxicity, through induction of oxidative stress and/or inflammation [7, 8].

*Caenorhabditis elegans* is a classic non-mammalian model animal [9]. Meanwhile, largely due to its sensitivity to environmental toxicants [10–12], *C. elegans* has already been widely used for toxicity assessment and toxicological study of various environmental toxicants, including carbon-based ENMs [13–17]. In nematodes, MWCNTs exposure could cause the damage on the functions of both primary targeted organs (such as intestine) and secondary targeted organs (such as neurons and reproductive organs) [18–21]. After exposure, MWCNTs could be potentially translocated into the secondary targeted organs, such as reproductive organs, through the intestinal barrier in nematodes [18, 21–23].

microRNAs (miRNAs) are a class of short noncoding RNAs, and can exhibit their biological functions by inhibiting the expression of targeted genes post-transcriptionally [24]. In nematodes, miRNAs have been shown to play important functions in regulating the toxicity of certain carbon-based ENMs, such as MWCNTs and graphene oxide (GO) [25–29]. For example, *mir-355* may regulate the MWCNTs toxicity by inhibiting the function of its target of DAF-2, an insulin receptor [28]. The activated *mir-259* may protect the nematodes from MWCNTs toxicity by inhibiting the function of its target of RSKS-1, a putative ribosomal protein S6 kinase [29]. In *C. elegans*, *let-7* is one of the founding members of the miRNA family firstly identified via forward genetic screen [30]. It has been shown that *let-7* regulates the timing of larval and adult transition by acting as a developmental switch, and the sequence and functions of *let-7* are conserved among different species [30, 31]. During the control of transition of developmental timing, *let-7* suppresses the expression and function of its direct targets of HBL-1 and LIN-41 [30, 31]. In this study, we investigated the potential effect of MWCNTs exposure on the molecular basis for developmental timing mediated by *let-7* and its targets of HBL-1 and LIN-41 using *in vivo* assay system of *C. elegans*. Our results demonstrated that the miRNA *let-7* could be decreased by MWCNTs exposure, and was required for the regulation of MWCNTs toxicity. *let-7* further regulated MWCNTs toxicity by suppressing the functions of HBL-1 signaling and LIN-41 signaling. Our data imply that the *let-7*-mediated molecular basis for the control of developmental-timing transition is dysregulated by MWCNTs exposure, and this molecular basis may be required for the control of MWCNTs toxicity in organisms.

## Results

### Effect of MWCNTs exposure on *let-7* expression

**Fig. 1:**
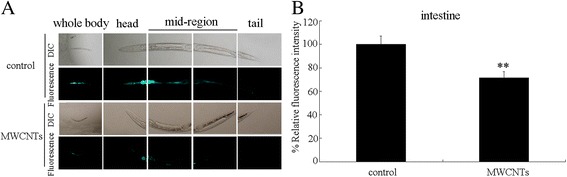
Effect of MWCNTs exposure on *let-7::GFP* expression. **a** Comparison of *let-7::GFP* expression in control and MWCNTs exposed nematodes. **b** Comparison of intestinal *let-7::GFP* expression in control and MWCNTs exposed nematodes. Thirty nematodes were examined per treatment. Prolonged exposure was performed from L1-larvae to young adults. Exposure concentration of MWCNTs was 10 μg/L. *Bars* represent means ± SD. ^**^ *P* < 0.01 vs control

Our previous studies have demonstrated that prolonged exposure (from L1-larvae to young adults) to MWCNTs at concentrations more than 1 μg/L could result in the significant induction of intestinal ROS production and decrease in locomotion behavior in nematodes [18, 21]. The concentration of 10 μg/L was selected as the working concentration for MWCNTs in this study. In *C. elegans*, *let-7* is expressed in almost all tissues except the germiline [32]. Using the transgenic strain of *zaEx5*[*let-7::GFP*], we observed that prolonged exposure to MWCNTs (10 μg/L) significantly decreased the *let-7::GFP* expression in the body of nematodes (Fig. 1a). We further focused on the intestinal cells to compare the relative fluorescence intensity of *let-7::GFP* between control and MWCNTs exposed nematodes. After MWCNTs (10 μg/L) exposure, the intestinal *let-7::GFP* was significantly decreased compared with control (Fig. 1b).

### Mutation of *let-7* induced a resistance to MWCNTs toxicity

**Fig. 2:**
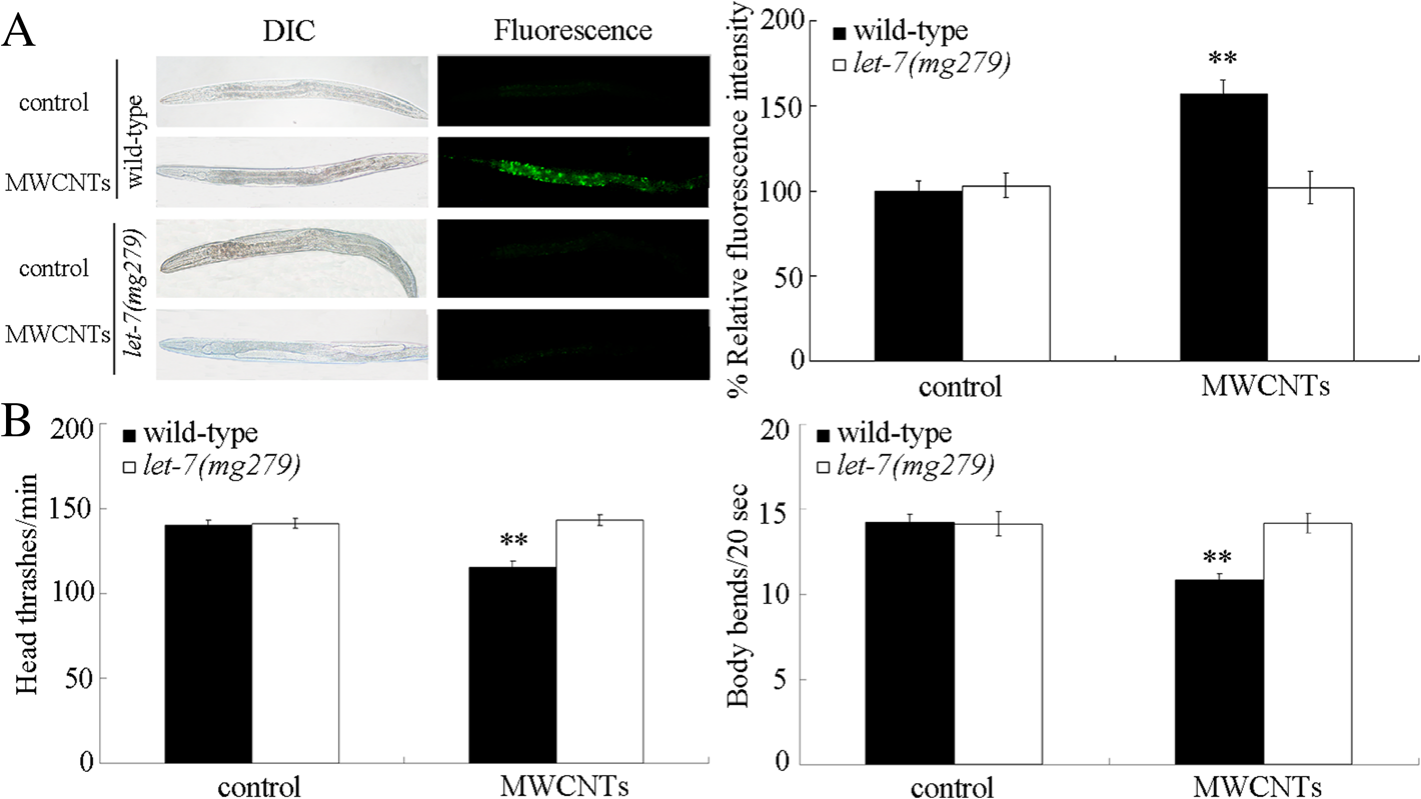
Effects of *let-7* mutation on MWCNTs toxicity. **a** Effects of *let-7* mutation on MWCNTs toxicity in inducing intestinal ROS production. Sixty nematodes were examined per treatment. **b** Effects of *let-7* mutation on MWCNTs toxicity in decreasing locomotion behavior. Sixty nematodes were examined per treatment. Prolonged exposure was performed from L1-larvae to young adults. Exposure concentration of MWCNTs was 10 μg/L. *Bars* represent means ± SD. ^**^ *P* < 0.01 vs control

Using the loss-of-function mutant of *let-7(mg279)*, we next investigated the effect of *let-7* mutation on the MWCNTs toxicity. Under normal conditions, loss-of-function mutation of *let-7* does not induce significant induction of intestinal reactive oxygen species (ROS) production and alteration in locomotion behavior (Fig. 2). After MWCNTs exposure, we found that the loss-of-function mutant of *let-7(mg279)* was resistant to MWCNTs toxicity in inducing intestinal ROS production and in decreasing locomotion behavior (Fig. 2). Therefore, mutation of *let-7* may induce a resistance to MWCNTs toxicity.

### *let-7* mutation increased the expressions of its targets of HBL-1 and LIN-41 in MWCNTs exposed nematodes

During the development, HBL-1 and LIN-41 have been identified as the direct targets for *let-7* in the control of developmental-timing transition [30, 31]. Under normal conditions, *let-7* mutation increases the expression of its targets of HBL-1 and LIN-41 [30, 31]. Moreover, *let-7* mutation significantly increased the expressions of *hbl-1* and *lin-41* in MWCNTs exposed nematodes (Additional file 1: Figure S1A). Additionally, MWCNTs could significantly increase the expressions of *hbl-1* and *lin-41* (Additional file 1: Figure S1B).

### Mutation of *hbl-1* or *lin-41* induced a susceptibility to MWCNTs toxicity

**Fig. 3:**
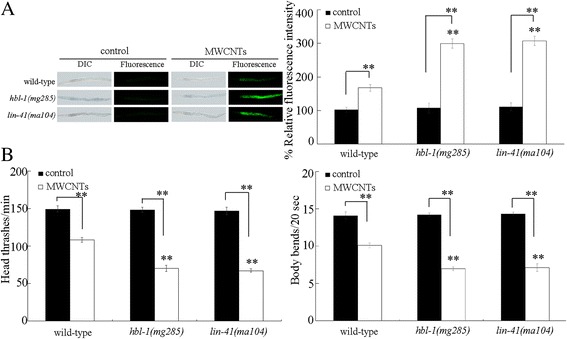
Effects of *hbl-1* or *lin-41* mutation on MWCNTs toxicity. **a** Effects of *hbl-1* or *lin-41* mutation on MWCNTs toxicity in inducing intestinal ROS production. Sixty nematodes were examined per treatment. **b** Effects of *hbl-1* or *lin-41* mutation on MWCNTs toxicity in decreasing locomotion behavior. Sixty nematodes were examined per treatment. Prolonged exposure was performed from L1-larvae to young adults. Exposure concentration of MWCNTs was 10 μg/L. *Bars* represent means ± SD. ^**^ *P* < 0.01 vs wild-type (if not specially indicated)

We further investigated the effect of *hbl-1* or *lin-41* mutation on MWCNTs toxicity. Under normal conditions, mutation of *hbl-1* or *lin-41* does not result in significant induction of intestinal ROS production and decrease in locomotion behavior (Fig. 3). Using intestinal ROS production and locomotion behavior as the endpoints, we found that mutation of *hbl-1* or *lin-41* caused the more severe MWCNTs toxicity in inducing intestinal ROS production and in decreasing locomotion behavior compared with those in MWCNTs exposed wild-type nematodes (Fig. 3). Therefore, mutation of *hbl-1* or *lin-41* may induce a susceptibility to MWCNTs toxicity.

### Genetic interaction between *let-7* and *hbl-1* or *lin-41* in the regulation of MWCNTs toxicity

**Fig. 4:**
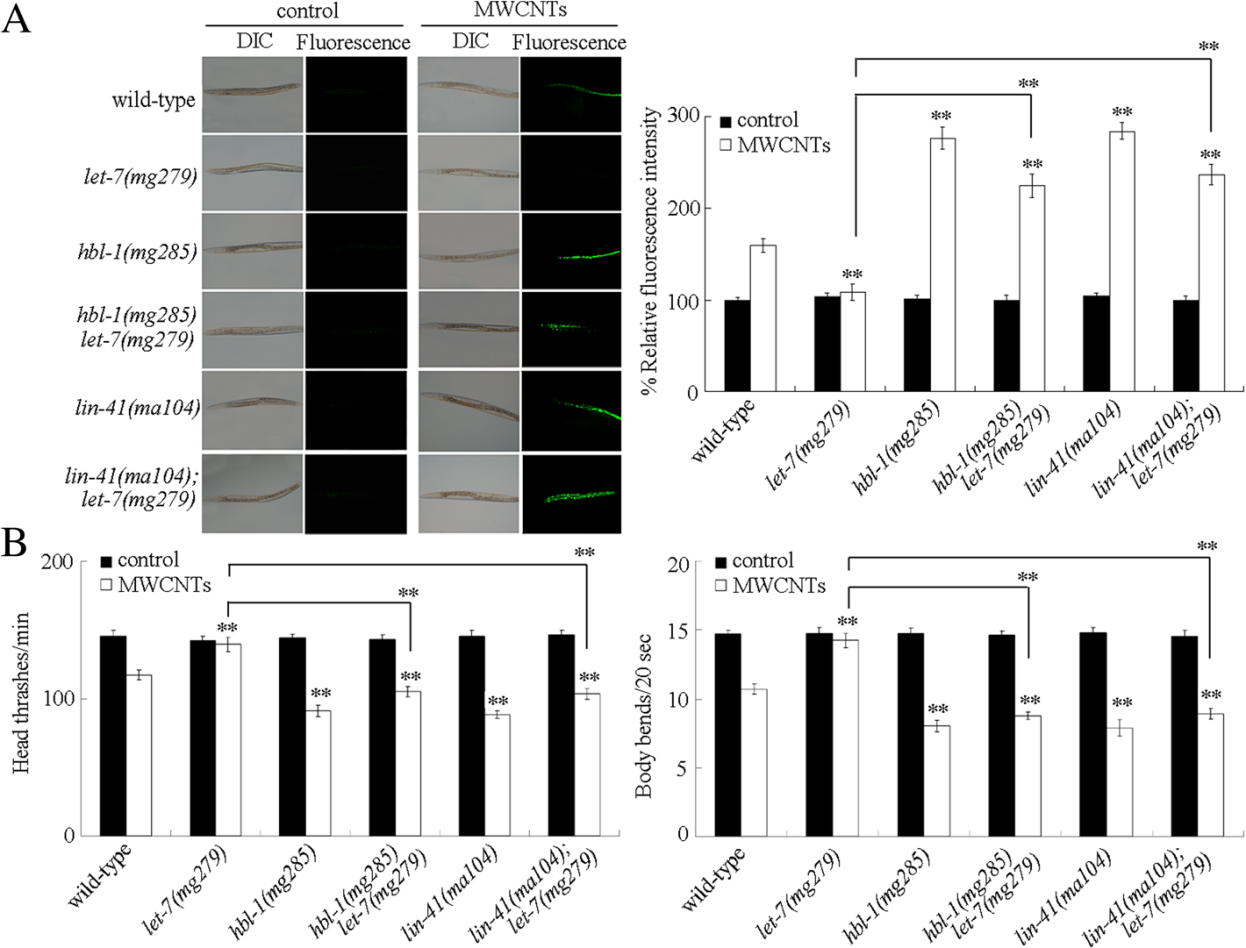
Genetic interaction between *let-7* and *hbl-1* or *lin-41* in the regulation of MWCNTs toxicity. **a** Genetic interaction between *let-7* and *hbl-1* or *lin-41* in the regulation of MWCNTs toxicity in inducing intestinal ROS production. Sixty nematodes were examined per treatment. **b** Genetic interaction between *let-7* and *hbl-1* or *lin-41* in the regulation of MWCNTs toxicity in decreasing locomotion behavior. Sixty nematodes were examined per treatment. Prolonged exposure was performed from L1-larvae to young adults. Exposure concentration of MWCNTs was 10 μg/L. *Bars* represent means ± SD. ^**^ *P* < 0.01 vs wild-type (if not specially indicated)

To further confirm the role of *let-7* and its targets in regulating MWCNTs toxicity, we next investigated the genetic interaction between *let-7* and *hbl-1* or *lin-41* in the regulation of MWCNTs toxicity. Under normal conditions, the double mutants of *hbl-1(mg285)let-7(mg279)* and *lin-41(ma104);let-7(mg279)* did not show a significant induction of intestinal ROS production and an obvious alteration in locomotion behavior (Fig. 4). After MWCNTs exposure, mutation of *hbl-1* or *lin-41* could effectively cause the significant induction of intestinal ROS production and decrease in locomotion behavior in *let-7(mg279)* mutant nematodes (Fig. 4). That is, mutation of *hbl-1* or *lin-41* could effectively suppress the resistance of *let-7(mg279)* mutant to MWCNTs toxicity in inducing intestinal ROS production and in decreasing locomotion behavior.

### Identification of downstream targets for HBL-1 in the regulation of MWCNTs toxicity

**Fig. 5:**
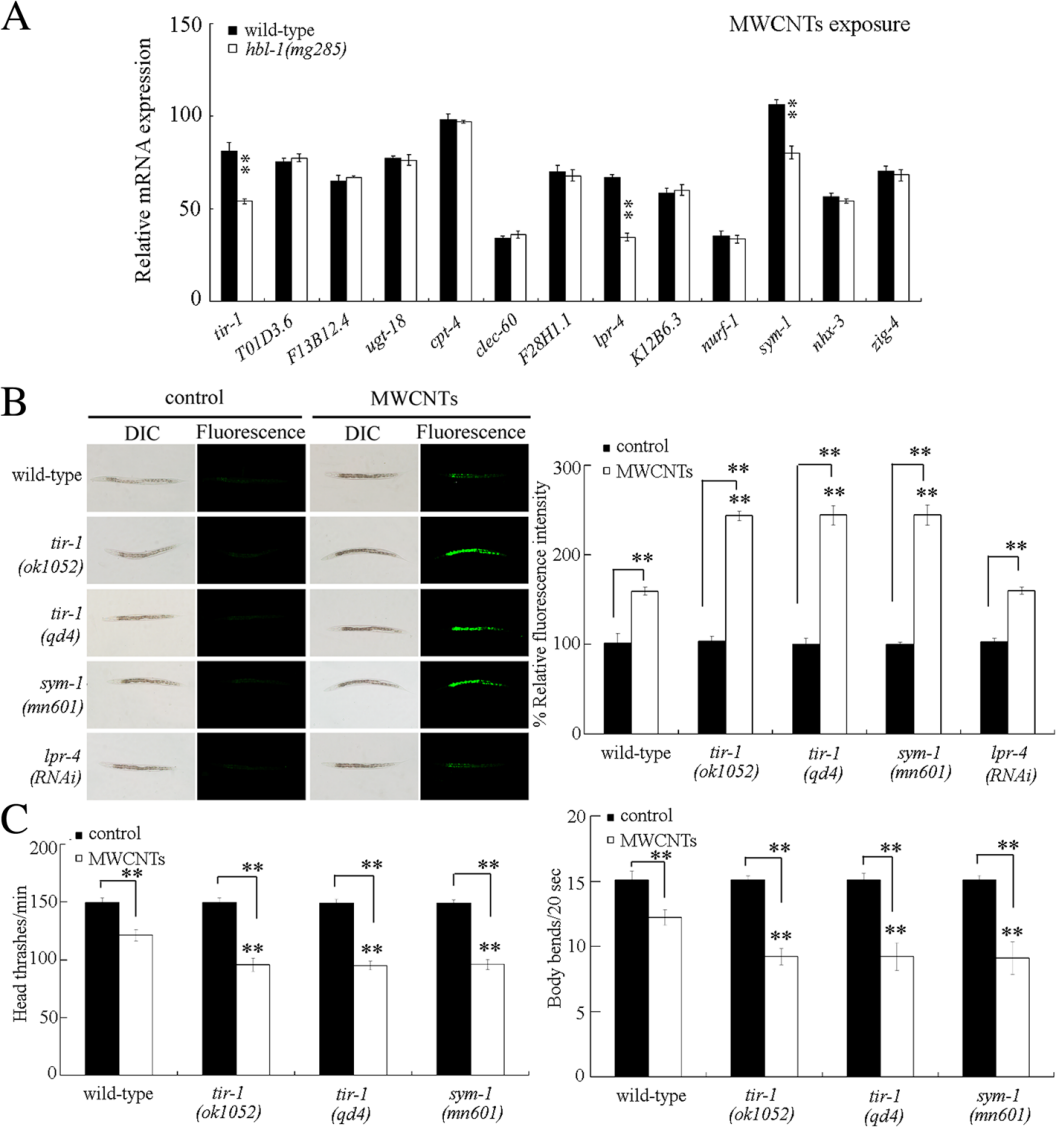
Identification of downstream targets of HBL-1 in the regulation of MWCNTs toxicity. **a** Effect of *hbl-1* mutation on the expressions of some potential target genes in MWCNTs exposed nematodes. **b** Effect of *tir-1* or *sym-1* mutation or RNAi knockdown of *lpr-4* on MWCNTs toxicity in inducing intestinal ROS production. Sixty nematodes were examined per treatment. **c** Effect of *tir-1* or *sym-1* mutation on MWCNTs toxicity in decreasing locomotion behavior. Sixty nematodes were examined per treatment. Prolonged exposure was performed from L1-larvae to young adults. Exposure concentration of MWCNTs was 10 μg/L. *Bars* represent means ± SD. ^**^ *P* < 0.01 vs wild-type (if not specially indicated)

In *C. elegans*, totally 13 genes (*T01D3.6*, *F13B12.4*, *ugt-18*, *cpt-4*, *clec-60*, *F28H1.1*, *W04G3.3*, *K12B6.3*, *nurf-1*, *sym-1*, *tir-1*, *nhx-3*, and *zig-4*) were significantly increased (more than 2.5 fold changes) by *hbl-1* overexpression under normal conditions [33]. After MWCNTs expression, the expressions of *tir-1*, *sym-1*, and *lpr-4* were significantly decreased by *hbl-1* mutation (Fig. 5a). In *C. elegans*, *tir-1* encodes a Toll-interleukin 1 receptor (TIR) domain adaptor protein, *sym-1* encodes a protein containing 15 contiguous leucine-rich repeats (LRRs), and *lpr-4* encodes a Lipocalin-related protein.

In MWCNTs exposed nematodes, mutation of *tir-1* or *sym-1* resulted in the more severe induction of intestinal ROS production compared with that in MWCNTs exposed wild-type nematodes (Fig. 5b). In contrast, mutation of *lpr-4* did not significantly affect the MWCNTs toxicity in inducing intestinal ROS production (Fig. 5b). Moreover, mutation of *tir-1* or *sym-1* led to the more severe decrease in locomotion behavior in MWCNTs exposed nematodes compared with that in MWCNTs exposed wild-type nematodes (Fig. 5c). These results suggest that mutation of *tir-1* or *sym-1* may induce a susceptibility to MWCNTs toxicity.

**Fig. 6:**
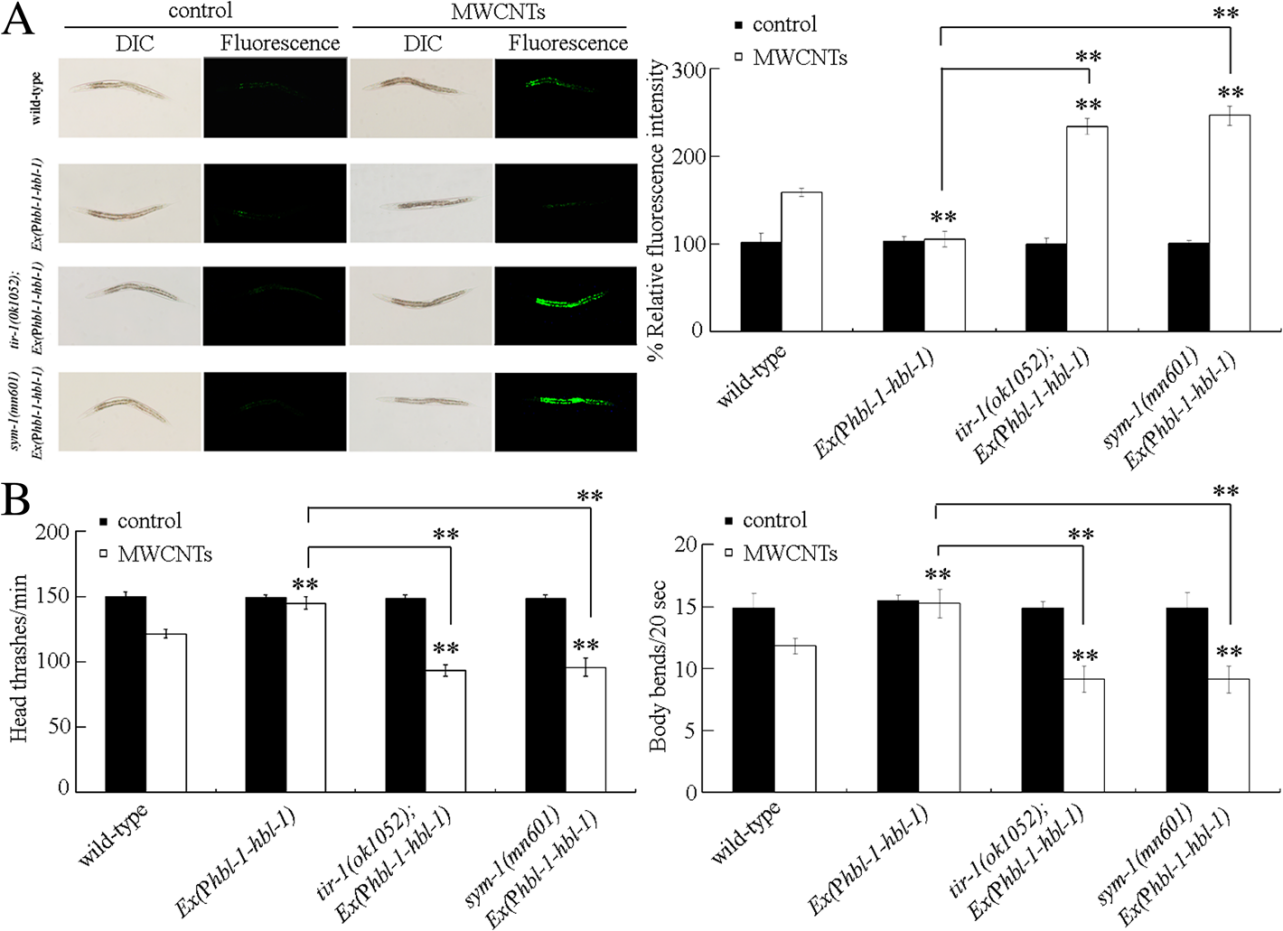
Genetic interaction between HBL-1 and TIR-1 or SYM-1 in the regulation of MWCNTs toxicity. **a** Genetic interaction between HBL-1 and TIR-1 or SYM-1 in the regulation of MWCNTs toxicity in inducing intestinal ROS production. Sixty nematodes were examined per treatment. **b** Genetic interaction between HBL-1 and TIR-1 or SYM-1 in the regulation of MWCNTs toxicity in decreasing locomotion behavior. Sixty nematodes were examined per treatment. WT, wild-type. Prolonged exposure was performed from L1-larvae to young adults. Exposure concentration of MWCNTs was 10 μg/L. *Bars* represent means ± SD. ^**^ *P* < 0.01 vs wild-type (if not specially indicated)

To further investigate the genetic interaction between HBL-1 and TIR-1 or SYM-1 in the regulation of MWCNTs toxicity, we generated the transgenic strain of *Ex(*P*hbl-1-hbl-1)* overexpressing HBL-1 in nematodes. The transgenic strain of *Ex(*P*hbl-1-hbl-1)* was resistant to MWCNTs toxicity in inducing intestinal ROS production and in decreasing locomotion behavior (Fig. 6). Moreover, we found that mutation of *tir-1* or *sym-1* significantly increased the induction of intestinal ROS production and decreased the locomotion behavior in MWCNTs exposed transgenic strain of *Ex(*P*hbl-1-hbl-1)* (Fig. 6). Therefore, mutation of *tir-1* or *sym-1* may suppress the resistance of transgenic strain of *Ex(*P*hbl-1-hbl-1)* to MWCNTs toxicity.

### Genetic interaction between LIN-41 and ALG-1 or ALG-2 in the regulation of MWCNTs toxicity

**Fig. 7:**
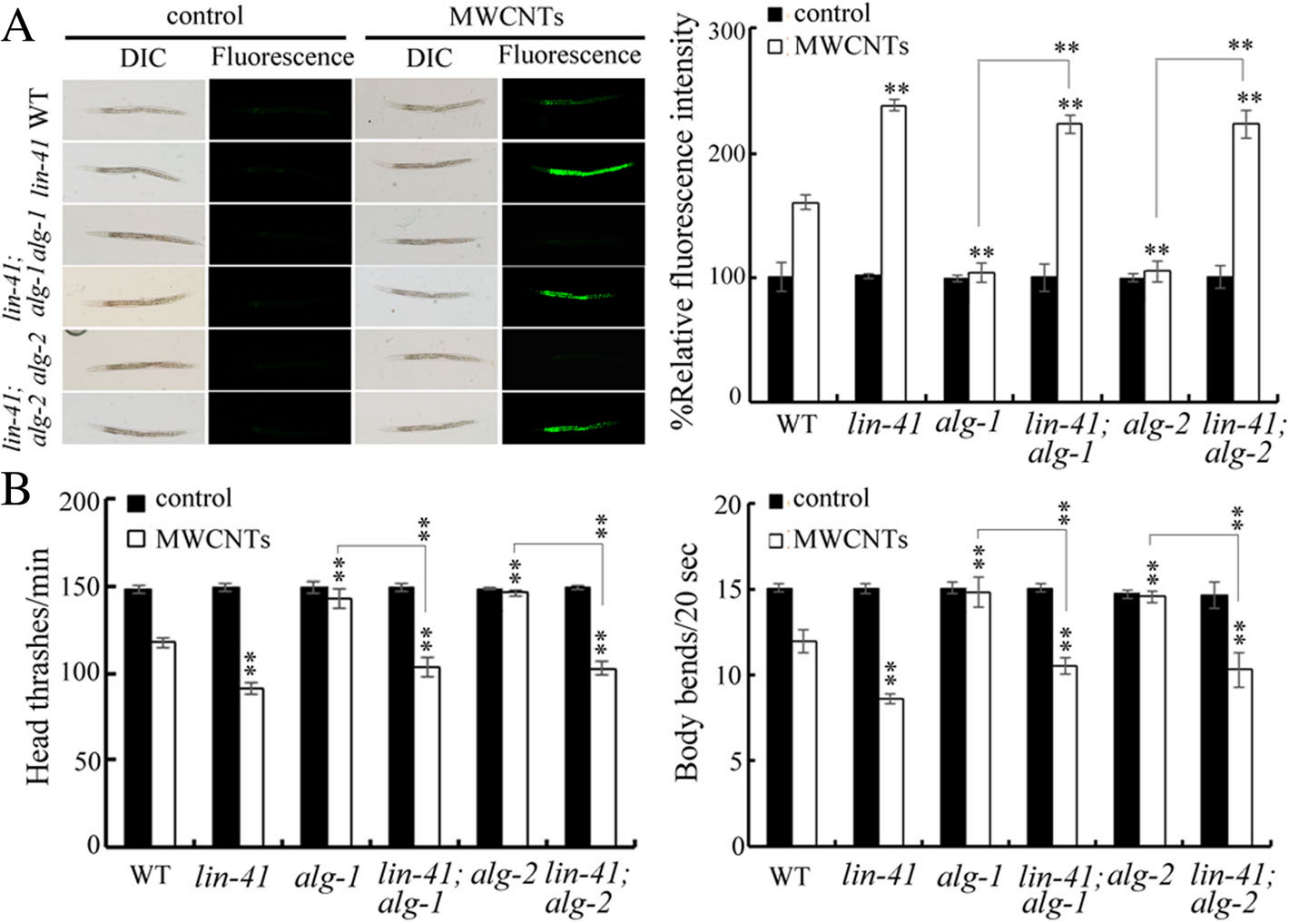
Genetic interaction between LIN-41 and ALG-1 or ALG-2 in the regulation of MWCNTs toxicity. **a** Genetic interaction between LIN-41 and ALG-1 or ALG-2 in the regulation of MWCNTs toxicity in inducing intestinal ROS production. Sixty nematodes were examined per treatment. **b** Genetic interaction between LIN-41 and ALG-1 or ALG-2 in the regulation of MWCNTs toxicity in decreasing locomotion behavior. Sixty nematodes were examined per treatment. WT, wild-type. Prolonged exposure was performed from L1-larvae to young adults. Exposure concentration of MWCNTs was 10 μg/L. *Bars* represent means ± SD. ^**^ *P* < 0.01 vs wild-type (if not specially indicated)

In *C. elegans*, *alg-1* and *alg-2* encode two members of RDE-1 proteins, and *lin-41* mutations could inhibit the retarded heterochronic phenotypes caused by *alg-1*/*alg-2* RNA interference (RNAi) [34]. We further examined the genetic interaction between LIN-41 and ALG-1 or ALG-2 in the regulation of MWCNTs toxicity. After MWCNTs exposure, we found that mutation of *alg-1* or *alg-2* could induce a resistance to MWCNTs toxicity in inducing intestinal ROS production and in decreasing locomotion behavior (Fig. 7). Moreover, after MWCNTs exposure, *lin-41* mutation could significantly suppress the resistance of *alg-1(gk214)* or *alg-2(ok304)* mutant to MWCNTs toxicity in inducing intestinal ROS production and in decreasing locomotion behavior (Fig. 7). These results suggest that ALG-1 or ALG-2 may act upstream of LIN-41 in the regulation of MWCNTs toxicity.

### A feedback loop between *let-7* and HBL-1 or LIN-41 in the regulation of MWCNTs toxicity

**Fig. 8:**
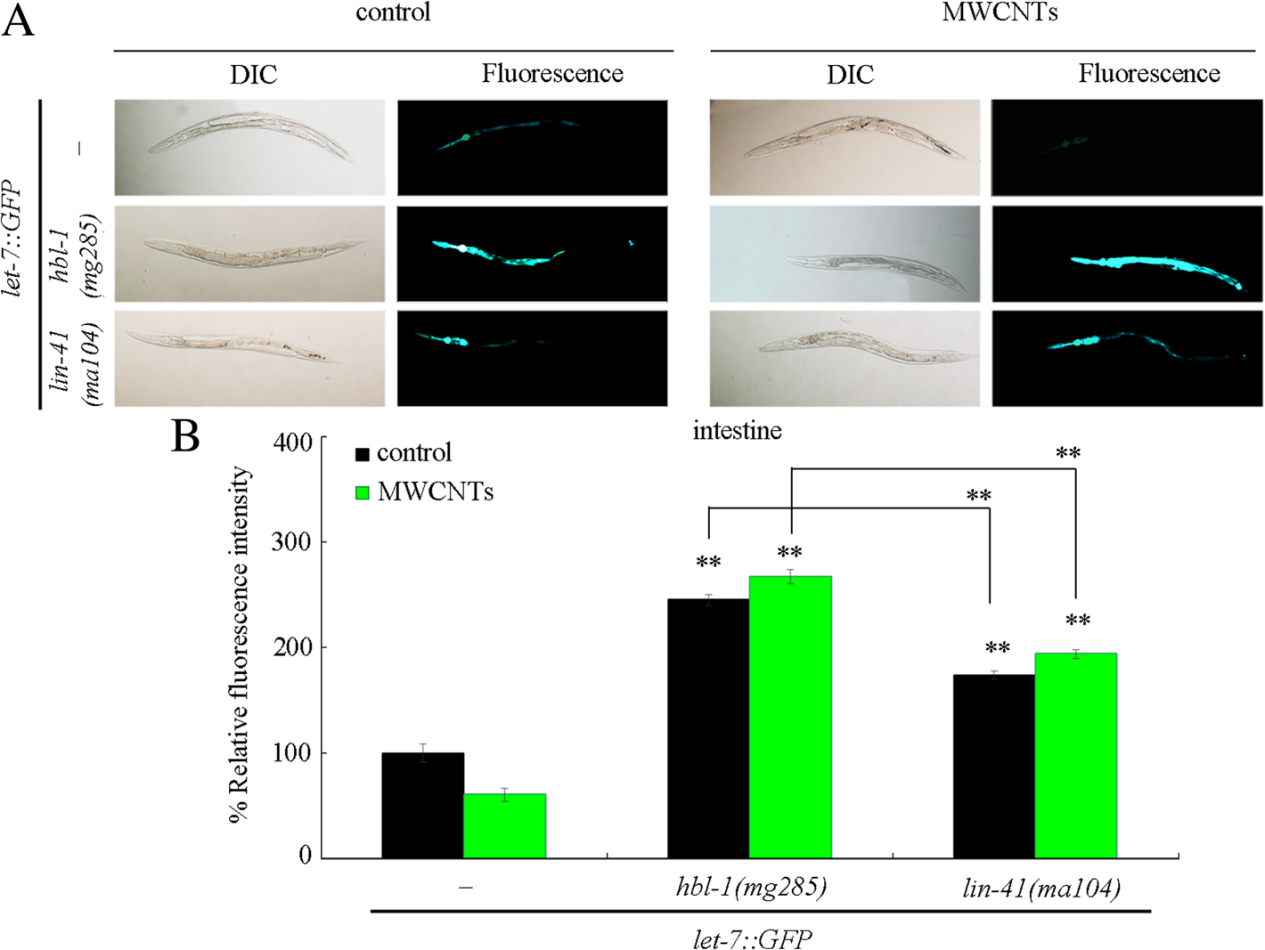
Effect of mutation of *hbl-1* or *lin-41* on *let-7::GFP* expression in MWCNTs exposed nematodes. **a** Comparison of *let-7::GFP* expression. **b** Comparison of intestinal *let-7::GFP* expression. “-”, nematodes without mutation of *hbl-1* or *lin-41*. Thirty nematodes were examined per treatment. Prolonged exposure was performed from L1-larvae to young adults. Exposure concentration of MWCNTs was 10 μg/L. *Bars* represent means ± SD. ^**^ *P* < 0.01 vs nematodes without mutation of *hbl-1* or *lin-41* (if not specially indicated)

It was reported that the *let-7* transcription can be temporally regulated by one of its targets, *hbl-1* [35]. We further found that, besides the *hbl-1*, mutation of *lin-41* also significantly increased the expression of *let-7::GFP* (Fig. 7). Mutation of *hbl-1* could cause the more significant increase in the expression of *let-7::GFP* compared with that in nematodes with mutation of *lin-41* (Fig. 8). Moreover, mutation of *hbl-1* or *lin-41* also significantly increased the expression of *let-7::GFP* in MWCNTs exposed nematodes (Fig. 8).

## Discussion

In this study, we examined the potential effect of MWCNTs exposure on the molecular basis for developmental timing using the *in vivo* assay system of *C. elegans*. In *C. elegans*, the miRNA *let-7* and its direct targets of HBL-1 and LIN-41 regulate the transition of developmental timing [30, 31]. After MWCNTs exposure, we observed the decreased expression of *let-7::GFP* (Fig. 1a). Moreover, we detected the increased expressions of *hbl-1* and *lin-41* in MWCNTs exposed nematodes (Additional file 1: Figure S1B). Therefore, MWCNTs may affect the molecular basis for developmental timing in animals.

Meanwhile, we investigated the potential role of *let-7* in the regulation of MWCNTs toxicity. We found that loss-of-function of *let-7* resulted in the formation of resistance to MWCNTs toxicity in inducing intestinal ROS production and in decreasing locomotion behavior (Fig. 2). Therefore, after MWCNTs exposure, the decreased *let-7* may mediate a protection mechanism for nematodes against the MWCNTs toxicity. More recently, it was also reported that *Pseudomonas aeruginosa* infection could suppress the *let-7* expression, and loss-of-function mutation of *let-7* enhanced the innate immune response of nematodes to *P. aeruginosa* infection [36, 37]. The induced decrease in *let-7* expression may be helpful for nematodes against the adverse effects from exposure to environmental toxicants or environmental pathogens.

For the normally identified targets of *let-7*, HBL-1 and LIN-41 [30, 31], we found that mutation of *hbl-1* or *lin-41* caused the formation of a susceptibility to MWCNTs in inducing intestinal ROS production and in decreasing locomotion behavior (Fig. 3). In *C. elegans*, *hbl-1* encodes a C2H2-type zinc finger transcriptional factor, and *lin-41* encodes a Ring finger-B box-Coiled coil (RBCC) protein. Both HBL-1 and LIN-41 are heterochronic proteins that play an essential role in the regulation of developmental timing during the postembryonic development [31, 38]. Our results imply that *let-7* and its targets may affect the MWCNTs toxicity by conferring a robust developmental timing control.

In *C. elegans*, *hbl-1* or *lin-41* translation is negatively regulated by the *let-7* that binds to the site(s) in the *hbl-1* 3’UTR or *lin-41* 3’UTR [30, 31, 38]. In this study, we further observed that both the *hbl-1* and the *lin-41* were increased by *let-7* mutation in MWCNTs exposed nematodes (Additional file 1: Figure S1A). Moreover, the resistance of *let-7(mg279)* mutant to MWCNTs toxicity in inducing intestinal ROS production and in decreasing locomotion behavior could be suppressed by *hbl-1* mutation or *lin-41* mutation (Fig. 4). Similarly, the resistance of *let-7* mutants to *P. aeruginosa* infection could also be suppressed by *hbl-1* mutation or *lin-41* mutation [37]. Therefore, the miRNA *let-7* may regulate both the MWCNTs toxicity and the innate immunity by suppressing the expressions and functions of its direct targets of HBL-1 and LIN-41.

**Fig. 9:**
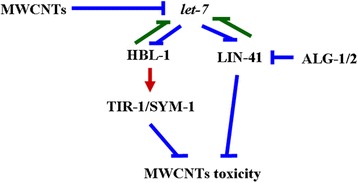
A diagram showing the molecular basis for *let-7* and its direst targets of HBL-1 and LIN-41 in the regulation of MWCNTs toxicity in nematodes

In *C. elegans*, TIR-1 and SYM-1 were identified as downstream targets for HBL-1 in the regulation of MWCNTs toxicity. On the one hand, under both normal and the MWCNTs exposure conditions, HBL-1 affects the activities of TIR-1 and SYM-1 (Fig. 5a) [33]. On the other hand, both *tir-1* mutant and *sym-1* mutant exhibited the similar susceptibility to MWCNTs toxicity to *hbl-1* mutant (Fig. 5b and c). Mutation of *tir-1* or *sym-1* also caused the enhanced susceptibility to pathogen infection [39, 40]. More importantly, we observed that mutation of *tir-1* or *sym-1* could suppress the resistance of transgenic strain overexpressing HBL-1 to MWCNTs toxicity (Fig. 6). Therefore, HBL-1 may regulate the MWCNTs toxicity by positively affecting the TIR-1 or SYM-1 activity. That is, a signaling cascade of HBL-1-TIR-1/SYM-1 was raised to be required for the regulation of MWCNTs toxicity (Fig. 9).

The gene-silencing phenomenon of RNAi is triggered by double-stranded (ds) RNA, and guides the mRNA destructio**n** [41]. In *C. elegans*, *alg-1* and *alg-2* are two genes related to the RNAi pathway [34]. In this study, we found that *alg-1* or *alg-2* mutation induced a resistance to MWCNTs toxicity in inducing intestinal ROS production and in decreasing locomotion behavior (Fig. 7), implying the further involvement of molecular basis for RNAi in the regulation of MWCNTs toxicity. Moreover, we observed that *lin-41* mutation suppressed the resistance of *alg-1* or *alg-2* mutant to MWCNTs toxicity (Fig. 7), indicating that LIN-41 may also regulate the MWCNTs toxicity by acting as the downstream target of ALG-1 or ALG-2 (Fig. 9). The genetic assay has further indicated the genetic suppression of *alg-1/alg-2(RNAi)* by *lin-41* in regulating the heterochronic phenotypes [34].

In this study, we also observed that both HBL-1 and LIN-41, the *let-7*’s direct targets, are responsible for the inhibition of transcription of *let-7*. Moreover, we found that mutation of *hbl-1* or *lin-41* significantly increased the expression of *let-7::GFP* in MWCNTs exposed nematodes (Fig. 8), suggesting a feedback loop may be formed between *let-7* and its targets of HBL-1 and LIN-41 in the regulation of MWCNTs toxicity. That is, in MWCNTs exposed nematodes, the suppressed *let-7* could be further inhibited by its targets of HBL-1 and LIN-41 (Fig. 9). Such a feedback mechanism will further strengthen the function of *let-7* suppression in protecting the animals from the MWCNTs toxicity in animals.

## Conclusions

In conclusion, we here investigated the potential effect of MWCNTs exposure on the molecular basis for developmental timing using *in vivo* assay system of *C. elegans*. We found that MWCNTs exposure could dysregulate the molecular basis for developmental timing as indicated by the altered expression patterns of *let-7* and its targets in MWCNTs exposed nematodes. Meanwhile, we observed the resistance of *let-7* mutant and the susceptibility of *hbl-1* or *lin-41* mutant to MWCNTs toxicity, suggesting the potential protection function of *let-7* suppression in being against the MWCNTs toxicity. We further found the formation of a feedback loop between *let-7* and its targets of HBL-1 and LIN-41 in the regulation of MWCNTs toxicity, which will further strengthen the function of *let-7* suppression in protecting the nematodes from the MWCNTs toxicity. Moreover, we identified the signaling cascades of HBL-1-TIR-1/SYM-1 and ALG-1/2-LIN-41 in the regulation of MWCNTs toxicity. Our results highlight the important link between the molecular basis for developmental timing and the regulation of MWCNTs toxicity in organisms.

## Methods

### Characterization of MWCNTs

MWCNTs (diameter: 10–20 nm, length: 5–15 μm) were from Shenzhen Nanotech. Port Co. Ltd. (Shenzhen, China). MWCNTs morphology in K-medium was examined by transmission electron microscopy (TEM, JEM-200CX, JEOL, Japan) (Additional file 1: Figure S2A). Length distribution of MWCNTs was shown in Additional file 1: Figure S2B based on the TEM assay. The presence of Ni (0.077%) and Fe (0.017%) impurities was determined by elemental inductively coupled plasma mass spectrometer (ICPMS). After prolonged exposure, it was found that both 0.077% Ni and 0.017% Fe in 1000 μg/L MWCNTs could not induce the toxicity, such as the induction of intestinal ROS production, on nematodes [21]. Zeta potential of MWCNTs was analyzed by Nano Zetasizer (Nano ZS90, Malvern Instrument, UK). Zeta potential of MWCNTs (1 mg/L) in K-medium was −32.9 ± 2.9 mV.

### *C. elegans* Strains and culture

Nematodes used in this study were wild-type N2, mutants of MT7626/*let-7(mg279)*, CT11/*hbl-1(mg285)*, CT8/*lin-41(ma104)*, RF54/*alg-1(gk214)*, RB574/*alg-2(ok304)*, RB1085/*tir-1(ok1052)*, ZD101*/tir-1(qd4)*, SP2163/*sym-1(mn601)*, *hbl-1(mg285)let-7(mg279)*, *lin-41(ma104);alg-1(gk214)*, *lin-41(ma104);alg-2(ok304)*, and *lin-41(ma104);let-7(mg279)*, and transgenic strains of CT12/*zaEx5*[*let-7::GFP*], *lin-41(ma104);zaEx5*, *Ex(*P*hbl-1-hbl-1)*, *tir-1(ok1052)*;*Ex(*P*hbl-1-hbl-1)*, *sym-1(mn601)Ex(*P*hbl-1-hbl-1)*, and *hbl-1(mg285)zaEx5*. Some of these strains were from *Caenorhabditis* Genetics Center (funded by NIH Office of Research Infrastructure Programs (P40 OD010440)). Nematodes were maintained on nematode growth medium (NGM) plates seeded with *Escherichia coli* OP50 at 20 °C [9], and lysed with a bleaching mixture (0.45 M NaOH, 2% HOCl) after washing off the plates into the centrifuge tubes. The age synchronous L1-larvae population was prepared as described previously [42].

### Exposure and toxicity assessment

MWCNTs were dispersed in K medium to prepare a stock solution (1 mg/mL). The stock MWCNTs solution was further sonicated for 30 min (40 kHz, 100 W), and diluted to the working concentrations (10 μg/L) with K medium just prior to exposure. Prolonged exposure to MWCNTs was performed from L1-larvae to young adults in 12-well sterile tissue culture plates at 20 °C in the presence of food (OP50). After MWCNTs exposure, the examined nematodes were used for the toxicity assessment using endpoints of intestinal ROS production and locomotion behavior.

The endpoint of intestinal ROS production was used to reflect the functional state of intestinal cells [43]. Intestinal ROS production was analyzed as described previously [44, 45]. After exposure, the examined nematodes were transferred to 1 μM 5′,6′-chloromethyl-2′,7′-dichlorodihydro-fluorescein diacetate (CM-H_2_DCFDA; Molecular Probes) solution to incubate for 3 h in the dark. After labeling, the nematodes were mounted on a 2% agar pad for the observation and examination at 488 nm of excitation wavelength and 510 nm of emission filter under a laser scanning confocal microscope (Leica, TCS SP2, Bensheim, Germany). Relative fluorescence intensity of ROS signals in the intestine was semi-quantified and expressed as the relative fluorescence units (RFU). Sixty nematodes were examined per treatment.

The endpoint of locomotion behavior was used to reflect the functional state of motor neurons [46]. Head thrash and body bend were selected to evaluate the locomotion behavior. The head thrash and the body bend were analyzed under a dissecting microscope by eyes as described previously [47, 48]. In *C. elegans*, a head thrash is defined as a change in the direction of bending at the mid body, and a body bend is defined as a change in the direction of the part of the nematodes corresponding to the posterior bulb of the pharynx along the *y* axis, assuming that nematode was traveling along the *x* axis. Sixty nematodes were examined per treatment.

### Reverse-transcription and quantitative real-time polymerase chain reaction (qRT-PCR) assay

Total RNAs of nematodes were extracted using an RNeasy Mini kit (Qiagen), and reverse transcribed using a PrimeScript ™ RT reagent kit (Takara, Otsu, Shiga, Japan). After the cDNA synthesis, real-time PCR was performed using the SYBR Premix Ex Taq™ (Takara) for the amplification of certain PCR products. Real-time PCR was run at an optimized annealing temperature of 58 °C. The relative quantification of targeted genes in comparison to a reference *tba-1* gene, encoding a tubulin, was determined. The final results were expressed as relative expression ratio between the targeted genes and the reference gene. All the reactions were performed for three biological replicates. For each biological replicate, three technical replicates were performed further. The related primer information for qRT-PCR is shown in Additional file 1: Table S1.

### RNAi assay

RNAi was performed by feeding nematodes with *E. coli* strain HT115 (DE3) expressing double-stranded RNA that is homologous to a target gene as described [49]. *E. coli* HT115 (DE3) was prepared onto NGM containing ampicillin (100 μg/mL) and isopropyl 1-thio-β-D-galactopyranoside (IPTG, 5 mM). L1 larvae were transferred onto RNAi plates for 2 days at 20 °C until they became gravid. Gravid adults were transferred onto the fresh RNAi-expressing bacterial lawns to lay eggs so as to obtain the second generation of RNAi population.

### DNA constructs and transformation

Promoter region for *hbl-1* amplified by PCR from wild-type *C. elegans* genomic DNA was inserted into the pPD95_77 vector in the sense orientation. *hbl-1/F13D11.2b* cDNA was amplified by PCR, and inserted into corresponding entry vector carrying the *hbl-1* promoter sequence. Germline transformation was performed as described by coinjecting a testing DNA (30 μg/mL) and a marker DNA (P*dop-1::rfp*, 60 μg/mL) into the gonad of nematodes [50]. The related primer information for DNA constructions were shown in Additional file 1: Table S2.

### Statistical analysis

Data in this article were expressed as means ± standard deviation (SD). Statistical analysis was performed using SPSS 12.0 software (SPSS Inc., Chicago, USA). Differences between groups were determined using analysis of variance (ANOVA), and probability levels of 0.05 and 0.01 were considered statistically significant. Graphs were generated using Microsoft Excel software (Microsoft Corp., Redmond, WA).

## Additional file

Additional file 1: Figure S1. Expression of *hbl-1* and *lin-41* in nematodes. A) Effect of *let-7* mutation on expression of *hbl-1* and *lin-41* after MWCNTs exposure. Bars represent means ± SD. ^**^ *P* < 0.01 vs wild-type. B) Effect of MWCNTs exposure on expression of *hbl-1* and *lin-41*. Bars represent means ± SD. ^**^ *P* < 0.01 vs control. Prolonged exposure was performed from L1-larvae to young adults. Exposure concentration of MWCNTs was 10 μg/L. **Figure S2.** Physiochemical properties of MWCNTs. A) TEM of MWCNTs after sonication. B) Length distribution of MWCNTs after sonication. **Table S1.** Primer information for qRT-PCR. **Table S2.** Primer information for DNA constructions. (DOC 204 kb)
