# Supplementary material for: Multi-walled carbon nanotubes-induced alterations in microRNA let-7 and its targets activate a protection mechanism by conferring a developmental timing control
Source: Part Fibre Toxicol. 2017 Jul 20;14:27. doi: 10.1186/s12989-017-0208-2 (PMC5520286; doi:10.1186/s12989-017-0208-2)
Supplement: Additional file 1: Figure S1. — Expression of hbl-1 and lin-41 in nematodes. A) Effect of let-7 mutation on expression of hbl-1 and lin-41 after MWCNTs exposure. Bars represent means ± SD. ** P < 0.01 vs wild-type. B) Effect of MWCNTs exposure on expression of hbl-1 and lin-41. Bars represent means ± SD. ** P < 0.01 vs control. Prolonged exposure was performed from L1-larvae to young adults. Exposure concentration of MWCNTs was 10 μg/L. Figure S2. Physiochemical properties of MWCNTs. A) TEM of MWCNTs after sonication. B) Length distribution of MWCNTs after sonication. Table S1. Primer information for qRT-PCR. Table S2. Primer information for DNA constructions. (DOC 204 kb) [file 12989_2017_208_MOESM1_ESM.doc]

**Supporting Information:**


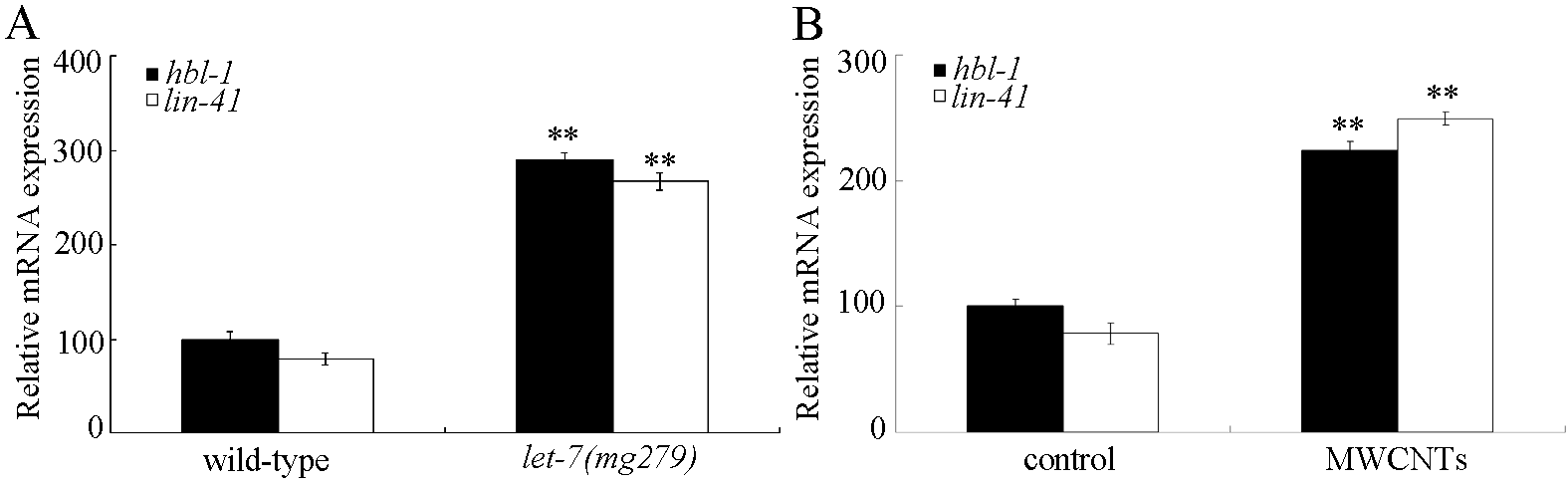


**Figure S1** **Expression of *hbl-1* and *lin-41* in nematodes.** **A)** Effect of *let-7* mutation on expression of *hbl-1* and *lin-41* after MWCNTs exposure. Bars represent means ± SD. ^**^*P* < 0.01 *vs* wild-type. **B)** Effect of MWCNTs exposure on expression of *hbl-1* and *lin-41*. Bars represent means ± SD. ^**^*P* < 0.01 *vs* control. Prolonged exposure was performed from L1-larvae to young adults. Exposure concentration of MWCNTs was 10 μg/L.


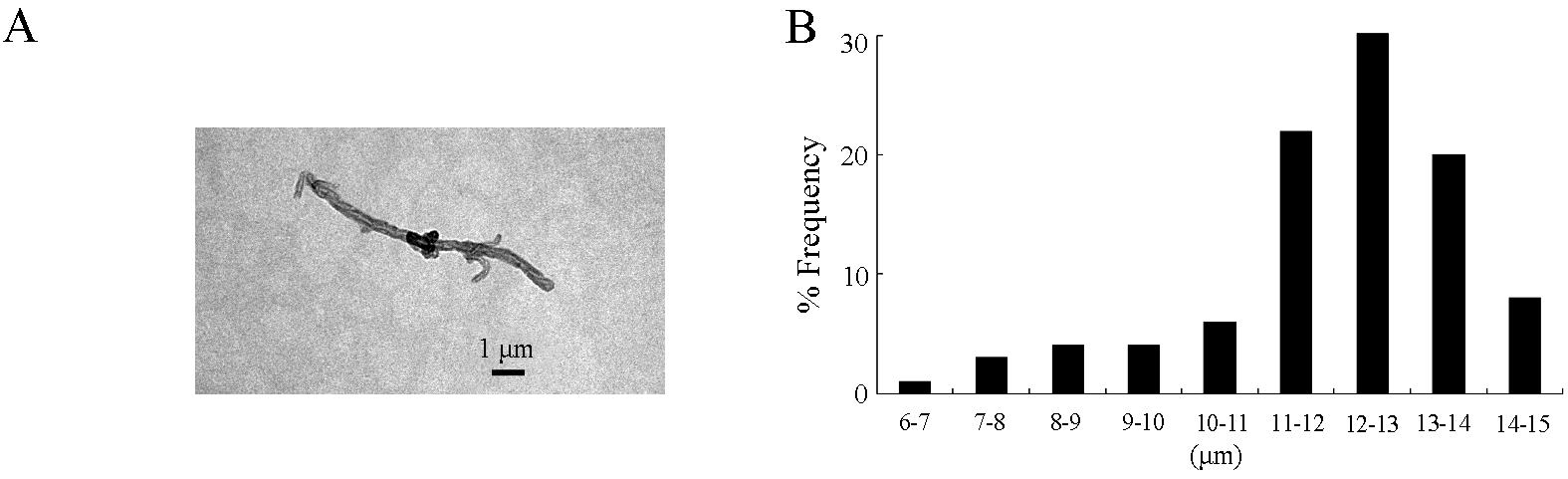


**Figure S2** **Physiochemical properties of MWCNTs. A)** TEM of MWCNTs after sonication. **B)** Length distribution of MWCNTs after sonication.

**Table S1** **Primer information for qRT-PCR**

| Gene | Forward primer (5’-3’) | Reverse primer (5’-3’) |
| --- | --- | --- |
| *hbl-1* | AAAGCCAGCCTGGAGGTTAC | GGCATCTGAACAGGCTCACT |
| *lin-41* | ACAACATGCGTTCGTTGAGC | GCCAGACACATGTCCTGAAA |
| *tir-1* | AAAGAGTTTGAGCCACTG | CTGCTCCTTCTTAATAGTTG |
| *T01D3.6* | TAGTCTAGCCCAGAGTTGCG | TCCTGGTCTGTGAGCGTTTC |
| *F13B12.4* | AAGCAAAATGGAGGCTCGGA | GTTCTCGTAGCTGTGGCAGT |
| *ugt-18* | CCGTCTCCAGCTTTGCCTAA | TTATCAGTGCCGGTTTCCCC |
| *cpt-4* | ATTGTGGCAGAGGTGATGGG | TGGGGAATGTTCTCCCGTTG |
| *clec-60* | CTGCAGTCTGGATGGCAGAA | CGCAAGATGCCGTTTCACAA |
| *F28H1.1* | AAGCAGAGTCGACAACAGCA | TGATAGATGGCGAACGGCTC |
| *lpr-4* | CAATTCTGCTGATGCCGAGC | TCTGAAGCGTTCTGGGTCAC |
| *K12B6.3* | GGCTTGTTTGCTCCCACTTG | ACTGGTCGATCGCTGTGTTT |
| *nurf-1* | TGAAGGCCCTACACGTCTTT | GCTGTCACACCCAACGTAGA |
| *sym-1* | GACAACGGAGTCTTGGAGCA | TCTAATTGGCCTTGGCTGGG |
| *nhx-3* | TGCTATGCAACAGCAGACGA | TGCAACCAAAAGCCATGTCG |
| *zig-4* | GGAGAAGCTTCGGGGTGTAG | TTTCGCGGTTGGGTAAAGGA |
| *alg-1* | TGCCGAACTGAACAACACCA | CAGGTAATTGTCCCGGTGCT |
| *alg-2* | TCCACCATGATCAAGGCCAC | CGCGTTTCACTTCGGCATAG |
| *tba-1* | TCAACACTGCCATCGCCGCC | TCCAAGCGAGACCAGGCTTCAG |

**Table S2 Primer information for DNA constructions**

| Gene | Forward primer (5’-3’) | Reverse primer (5’-3’) |
| --- | --- | --- |
| P*hbl-1* | TATCCCGGGAACGTTGTCTACCGCGCT | TATGGTACCCTGTTGTAACCTCCAGGC |
| *hbl-1* | ATAGGTACCATGCCTGTACAGCTCAGC | ATACCCGGGTTATTGGTGTCTGGCTTG |
